# International Multicenter Video Review on Neonatal Procedures: Lessons Learned from a Collaborative Study

**DOI:** 10.3390/children13060816

**Published:** 2026-06-13

**Authors:** Veerle Heesters, Hannah Schwarz, Henriette A. van Zanten, Katharina Bibl, Tobias Werther, Katrin Klebermass-Schrehof, Angelika Berger, Sophie Jansen, Arjan B. te Pas, Ruben Witlox, Michael Wagner

**Affiliations:** 1 Division of Neonatology, Department of Paediatrics, Willem-Alexander Children’s Hospital, Leiden University Medical Center, 2333 ZA Leiden, The Netherlands; h.a.van_zanten@lumc.nl (H.A.v.Z.); r.witlox@lumc.nl (R.W.); 2 Division of Neonatology, Department of Pediatrics and Adolescent Medicine, Comprehensive Center for Pediatrics, Medical University of Vienna, 1090 Vienna, Austriasophie.jansen@meduniwien.ac.at (S.J.); michael.b.wagner@meduniwien.ac.at (M.W.)

**Keywords:** video recording, neonatal care, quality improvement, video review, international

## Abstract

**Highlights:**

**What are the main findings?**

- Multicenter video review identifies critical procedural variations and facilitates international, interprofessional dialogue between neonatal centers.
- Collaborative video review generates novel clinical perspectives on care, informing possible refinement and adaptation of local guidelines at both institutions participating in multicenter video review.

**What are the implications of the main findings?**

- Expanding video review from a local to a multicenter framework uncovers hidden clinical disparities that standard guideline comparisons overlook.
- The provided implementation roadmap offers a strategy to foster international knowledge exchange and could potentially enhance patient safety on a broader scale.

**Abstract:**

**Background/Objectives:** The Leiden University Medical Center (LUMC) and the Medical University of Vienna (MUV) both implemented video recording and review in their neonatal intensive care unit (NICU). The two centers initiated collaborative, multicenter video review sessions to facilitate international knowledge exchange. **Methods**: In this exploratory, descriptive study, collaborative video review sessions were organized with the interprofessional NICU staff of the LUMC and the MUV. We aimed to describe our experience with organizing these sessions and to report procedural variations, and document lessons learned that led to new perspectives on care. **Results**: We conducted five sessions using recordings of different patients undergoing intubation, less invasive surfactant administration, umbilical, central-catheter insertion and physiologically based cord clamping after birth. The videos were selected to ensure technical and clinical comparability. Sessions were attended by a mean of eight providers per center. A total of 19 relevant differences were described, of which seven (37%) prompted changes in practice or new insights for one or both centers. Finally, we developed a roadmap for organizing multicenter video review sessions. **Conclusions**: This study shows that multicenter video review may represent a feasible and innovative educational approach for identifying practice variations and fostering cross-institutional clinical refinement.

## 1. Introduction

Video recording of real-time medical procedures has become a valuable tool for reflection in medical care. It provides an objective perspective on care delivery, facilitating interprofessional learning and quality improvement initiatives [1,2,3,4]. In the neonatal intensive care unit (NICU), video review has been applied to procedures such as endotracheal intubation, surfactant administration, umbilical catheter insertion, and neonatal resuscitation [5,6]. Both the Leiden University Medical Center (LUMC) and the Medical University of Vienna (MUV) have successfully integrated video recording and reviewing into their NICU practices [7,8].

Despite international neonatal procedural guidelines, variations in care persist, highlighting the need for ongoing analysis and refinement of practice [9,10,11,12,13]. Video recordings offer an effective means to observe these variations objectively. Previous research has demonstrated that video recordings can be used to identify significant differences in delivery room management across neonatal centers [14]. While traditional multicenter observational studies typically quantify such disparities retroactively, multicenter video review—whereby clinicians from different institutions collaboratively and dynamically analyze live-action recordings—could help providers gain new perspectives on their clinical practice. This enables centers to learn from one another and improve their respective practices. The aim of this study was to describe our experience with multicenter video review, report lessons learned from conducting multicenter review sessions on neonatal procedures and delineate the subsequent changes implemented in practice. To explicitly guide this evaluation, our secondary objectives were to assess the feasibility of this collaborative approach and to develop a roadmap for other institutions seeking to implement multicenter video review.

## 2. Materials and Methods

For this exploratory, descriptive multicenter video review study, we planned and conducted five collaborative video review sessions with neonatal care providers working in the NICUs of the LUMC (Leiden, the Netherlands) and the MUV, (Vienna, Austria). Both institutions are tertiary level perinatal centers that record interventions in the NICU using eye-tracking glasses and static video systems. Video review sessions were implemented in both centers several years prior and are part of the weekly clinical routine within the departments.

Both the LUMC and MUV utilized eye-tracking glasses: the LUMC used the Tobii Pro Glasses 3 (Tobii, Stockholm, Sweden), while the MUV also used the VPS 19 (ViewPoint System, Vienna, Austria) to record the visual field of the healthcare providers and provide a point-of-view recording [5]. Additionally, both centers used a stationary camera, either a GoPro (GoPro Inc, San Mateo CA, USA) or a Microsoft LifeCam Cinema (Microsoft Ireland Operations Ltd., Dublin, Ireland), to capture a wide-angle view of the patient’s bed or stabilization room. Video laryngoscopy (Acutronic, Hirzel, Switzerland or Verathon GlideScope, Bothell, WA, USA) recordings were integrated with the point-of-view recording where possible.

For this study, five collaborative, multicenter video review sessions were conducted, focusing on each of five different procedures: (1) endotracheal intubation; (2) minimally invasive surfactant therapy or less invasive surfactant administration (MIST/LISA); (3) umbilical venous catheter (UVC) insertion; (4) peripherally inserted central venous catheter (PICC) insertion; and (5) physiologically based cord clamping (PBCC) during stabilization at birth. These specific target topics were selected because they represent the most frequently performed and routinely recorded critical care interventions within both NICUs and were identified a priori as procedures where collaborative video review would yield the highest clinical and educational value. Each multicenter session included one video from each center. Two study coordinators (VH and HS) independently screened the local databases, which at the time contained a total pool of approximately one hundred recordings in total, of all eligible interventions, across both institutions. Both centers selected a representative video of the specific intervention from their respective databases based on technical comparability, recentness to reflect current standard practice, and consent from parents and caregivers. Videos were excluded if they had poor audio–visual quality or incomplete procedural documentation. Proceduralists, defined as the recorded providers involved in hands-on care, who either wore eye-tracking glasses or were present in the room during the recording of the procedure, gave oral consent for the educational and research use of the recording in multicenter video review. This clinician consent was video-specific; prior to each multicenter session, providers were explicitly informed which video would be reviewed and were given the option to opt out without any consequences. Parental consent protocols differed between institutions: at the MUV, prospective parental consent was obtained prior to video recording, whereas in the LUMC, only the unidentifiable recordings in the stabilization room were part of standard clinical care for quality assurance purposes, and therefore exempt from patient consent under local institution guidelines. However, identifiable recordings made with the eye-tracking glasses or the GoPro camera were not part of standard clinical care, and therefore consent was obtained prior to video recording and for use of a video in multicenter video review. In addition, to prevent re-identification, all recordings were de-identified before review by digitally blurring visible patient features or editing the video to eliminate identifying data. The videos were not transferred, emailed, or exchanged as raw data files. Instead, the video data remained hosted on the native institution’s server and were shared only via real-time screen sharing during online meetings.

Audio was recorded, although due to language barriers not all conversations could be understood. Consequently, during the online meetings, a representative from each center introduced their case and provided contextual information, followed by a structured discussion to address cross-institutional questions. Furthermore, during the live online sessions, the respective native-speaking study coordinator provided immediate contextual translation and standardization of terminology to ensure accurate cross-institutional interpretation. Two researchers, one from each center (VH and HS), prepared and chaired each video review session. A variation in practice was classified as relevant if it was explicitly flagged by the participants during the collaborative review as having a potential clinical or educational impact during the (online) discussion. To ensure reproducibility and minimize subjective bias, these variations were discussed, verified, and agreed upon by consensus between the attending providers of care from both centers who participated in multicenter video review. In this context, consensus was defined a priori as a unanimous agreement among all session participants that a variation was relevant to discuss and if so, whether it would lead to a modification of practice in a center; if an objection was raised, this subject was debated further until agreement was reached.

To evaluate the multicenter video review process, three outcome measures were predefined:

(1). Roadmap development: Essential operational and technical requirements for conducting cross-border multicenter reviews were identified during the sessions and synthesized to develop a structured implementation roadmap.

(2). Procedural variations: The number of relevant variations in neonatal procedural practices between the two centers was identified and documented before the video review sessions and adjusted afterwards.

(3). Practice modifications: Insights informing potential modifications of existing clinical approaches were documented, based on their subsequent institutional impact.

## 3. Results

Five multicenter video review sessions were conducted between December 2022 and March 2024. The reviewed procedures included endotracheal intubation, MIST/LISA, UVC insertion, PICC insertion, and PBCC during stabilization following birth.

### 3.1. Roadmap Development

**Figure 1:**
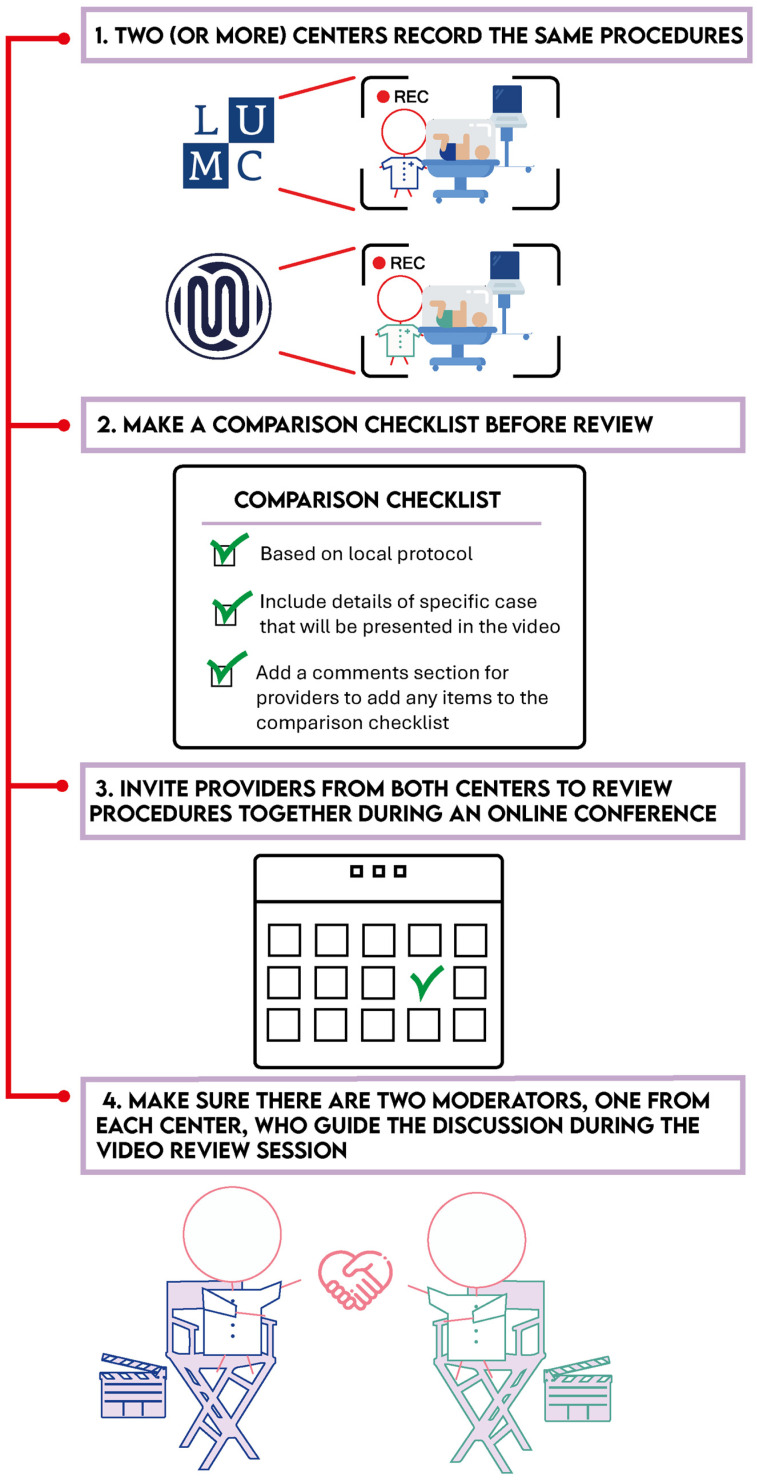
Roadmap for organizing multicenter video review sessions.

Based on our experience, a roadmap for conducting multicenter video review sessions was developed (Figure 1). This roadmap delineates the sequential steps required to establish and execute cross-border collaborative reviews.

1. Two centers that had independently established local video recording and review programs initiated a collaboration to organize multicenter video review and collaboratively review recordings of neonatal procedures. Importantly, both NICUs managed a similar patient population, ensuring that the evaluated neonatal procedures were clinically comparable.
2. Prior to each session, a specific procedure was selected and technically comparable recordings were identified. To guide the dialogue, a comparison checklist was created, based on the local guidelines of each center and distributed among attending staff members prior to the video review session (Appendix A). This checklist incorporated critical clinical and contextual characteristics, such as gestational age, birthweight and indication for the intervention. The protection of patient data was upheld through the anonymization of patient data in the videos, alongside the utilization of a sharing-only format for the online review sessions.
3. Providers from both centers, comprising both medical and nursing staff, were invited to collaboratively evaluate the procedure in an online meeting. Across the five sessions, attendance varied between 2 and 20 participants per session from each center, with a mean attendance of eight neonatal providers per session. On average, five neonatal care providers from the MUV and eleven from the LUMC attended the sessions. Sessions lasted between 30 and 60 min and were hosted via online video conferencing platforms (Cisco Systems, Inc., San Jose, CA, USA) or Microsoft Teams (Microsoft, Redmond, Washington, DC, USA).
4. Sessions were co-chaired by the researchers from both centers (VH and HS) to moderate the online session, monitor the time and maintain a safe learning environment. Following a brief introduction of the clinical case, providers viewed the recordings from both centers and engaged in open discussions and addressed cross-institutional queries. During the sessions, the baseline checklists were iteratively updated to integrate immediate lessons learned from the discussion (Appendix A). Throughout the study period, the researchers deliberated these lessons learned within their respective departments and documented subsequent practice modifications based on the multicenter sessions.

### 3.2. Procedural Variations

**Figure 2:**
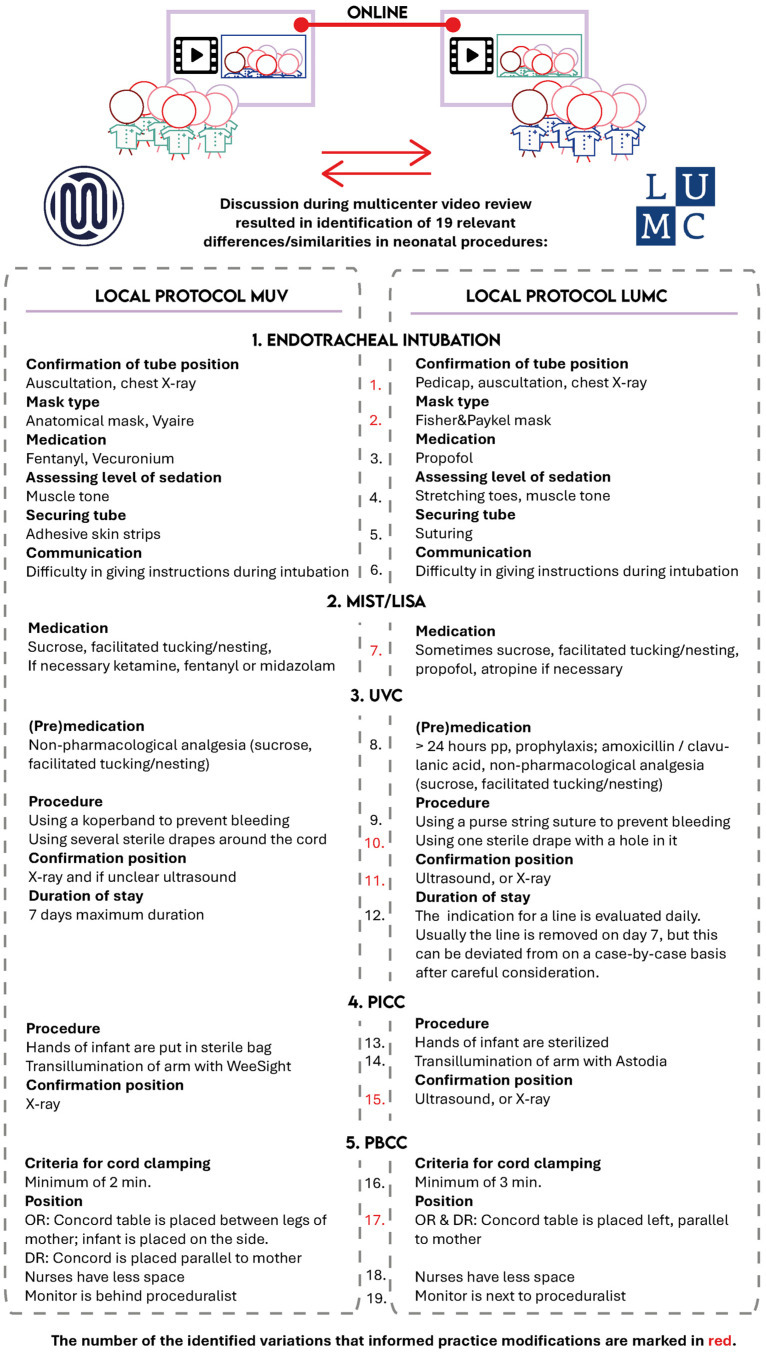
Description of the relevant differences in neonatal procedures identified during multicenter video review.

During the sessions, providers discussed the recorded procedures and iteratively refined the comparison checklists, resulting in the identification of 19 relevant procedural variations (Figure 2). In Table 1, these variations are described in more detail. Given the multiplicity of acceptable clinical strategies within the neonatal field, not all observed disparities between the two centers were deemed relevant through consensus between attending providers of care who participated in multicenter video review.

### 3.3. Practice Modifications

Of the 19 identified variations, 7 (37%) provided novel clinical insights that potentially warranted a modification of practice in one or both centers. The critical variations leading to these practice modifications are also described in Table 1. The five collaborative video review sessions yielded distinct procedural insights and drove targeted practice modifications across both institutions, spanning airway management, vascular access, and delivery room stabilization:

Session 1 (Intubation): Dialogue focused on pre-medication, tube fixation, and sedation depth indicators. Following the session, the MUV increased its use of colorimetric CO_2_ detectors for faster tube verification, while the LUMC adopted the MUV’s face mask models.

Session 2 (MIST/LISA): Discussions centered on sedation thresholds. Observing the MUV’s success with non-pharmacological positioning and sucrose prompted the LUMC to launch a targeted staff training program to optimize infant comfort and reduce sedative reliance.

Session 3 (UVC): Focus areas included sterility and line localization. To improve sterility, the LUMC adopted a novel cord-cutting technique and the MUV updated its clamp-draping checklists. Additionally, the LUMC’s routine use of ultrasound for tip localization motivated the MUV to initiate junior faculty ultrasound training and establish new clinical guidelines.

Session 4 (PICC): Variations were identified in sterile field preparation and anatomical depth measurements. The session fostered a cross-institutional dialogue on team training requirements for implementing operator-dependent ultrasonography for line verification.

Session 5 (PBCC): Review of this new technique highlighted logistical challenges in trolley positioning, infant placement, and clamping times. These insights led the LUMC to integrate the MUV’s room configuration into its implementation strategy, while both centers used the session to optimize nursing roles and equipment access.

## 4. Discussion

Previous experience in conducting local video review prompted our institutions, the MUV and the LUMC, to organize multicenter video review sessions to elevate reflection on clinical practice [7,8]. In this study, neonatal care providers identified 19 relevant procedural variations and fostered international knowledge exchange. Interprofessional discussions and real-time visualization of these variations provided healthcare providers with alternative perspectives on their performance, prompting a re-evaluation of care and discussions regarding the modification of established aspects of neonatal procedures in 37% (7/19) of the identified variations. While previous cross-center video comparisons have typically been limited to researcher-driven analysis, this exploratory study describes an international, collaborative approach utilizing transparent, interprofessional evaluation [14]. Our experience suggests that multicenter video review has the potential to serve as a useful tool for shared learning and collaborative practice reflection across participating centers, confirming the implications of research on video review in other specialties. Garcia et al. shows that video review has the potential to optimize team dynamics and enhance safety during emergent endotracheal intubations [15].

Neonatal care is often compared internationally by evaluating local guidelines using surveys or chart reviews [16,17,18,19,20,21]. However, retrospective documentation can be limited by recall bias, and guidelines may lack clinical realism or strict adherence [4,22]. Real-time recording of medical care provides a more objective view of actual practice, enabling providers to discuss procedural nuances and “grey areas” not captured in written protocols [3,10,23]. Our findings support this, as we identified interhospital variations that had not been detected during a preliminary review of local guidelines, such as the varying focus on non-pharmacological comfort measures during MIST/LISA. Furthermore, differences in analgesia used for intubation reflect existing gaps in the literature regarding the risks and benefits of specific sedative medications [10]. Although consensus on a single best approach was not reached, these variations present an opportunity to further study the relative effectiveness of different methods [24].

The collaborative sessions also served as a platform to discuss the introduction of new techniques. For example, both centers shared experiences regarding the ongoing implementation of point-of-care ultrasonography (POCUS) for central line localization, a modality that can, in certain clinical scenarios, reduce reliance on standard X-ray imaging [25,26]. Similarly, discussions regarding physiologically based cord clamping (PBCC) highlighted minor variations in infant wrapping, table placement, and minimum clamping times. Reviewing these variations allowed clinicians to share logistical solutions derived from their respective environments (the delivery room versus the operating room), illustrating how multicenter dialogue may streamline the future introduction of novel equipment or techniques.

While the real-time visualization utilized in this study shares conceptual elements with telemedicine, our approach focused strictly on collaborative reflection to allow for a thorough evaluation of completed procedures [27,28,29]. In the future, multicenter video review might theoretically reinforce active telemedicine networks by providing a framework to debrief past clinical exchanges, or help bridge knowledge gaps in lower-resource settings where camera equipment is available. However, these broader applications remain speculative and require formal evaluation.

Several limitations must be acknowledged. First, because centers selected representative videos from their own databases, a degree of selection bias is inherent, as the recordings may overrepresent optimal performances rather than baseline practice. However, this bias was likely mitigated by the dynamic, interprofessional discussions; participants frequently provided verbal context during the sessions regarding whether the recorded actions reflected standard routine or exceptional circumstances. Second, some providers were already professionally acquainted, which may have facilitated an environment of mutual understanding. On the other hand, potential interpretive bias and circularity must be acknowledged, as the study coordinators (VH, HS) functioned as both session moderators and the primary evaluators responsible for documenting lessons learned. To minimize this bias, the analysis relied strictly on consensus-driven validation; lessons learned and practice modifications were only documented if they were explicitly raised and corroborated by the active clinical participants during the interprofessional discussions, rather than being retroactively deduced by the moderators. Furthermore, the use of a pre-established, standardized checklist ensured a structured evaluation process. Crucially, both centers were already highly experienced in local video review and accustomed to a safe, non-punitive feedback culture; consequently, these findings may not generalize to novice centers where video review is less established. Third, although sessions were conducted in English, potential language barriers may have subtly affected the comprehension of subtle audio nuances during the recorded procedures. Fourth, due to the exploratory, observational nature of this study across five distinct procedures, the lessons learned cannot be broadly generalized, and this study did not assess whether the discussed practice modifications were successfully sustained over time. Finally, organizing international sessions was highly time-consuming, resulting in a limited number of meetings and lower nursing staff attendance from the MUV due to scheduling constraints. Future research should include a wider array of procedures, utilize continuous checklist adaptations, and objectively measure the long-term clinical outcomes of video-driven practice modifications.

## 5. Conclusions

We described our experience with using real-time recordings of neonatal procedures for conducting multicenter video review to learn from international differences between NICUs. Our findings demonstrate that cross-border, interprofessional video review is a useful methodology that successfully identified critical procedural variations, informing adaptation of local guidelines in both centers. By providing a structured implementation roadmap, this study underscores the novelty and scalability of collaborative video analysis as a potential tool for global medical education and quality improvement initiatives. Ultimately, establishing such international networks opens new avenues for multicenter research and may contribute to patient-safety-oriented learning in neonatal intensive care.

## Figures and Tables

**Table 1:** Relevant identified variations and lessons learned from multicenter video review sessions that informed practice modifications.

	^1^	Practice Variations	Practice Modifications
Session 1: Endotracheal intubation	1.	The first topic of discussion was the use of a colorimetric CO_2_ detector (Pedi-Cap; Nellcor Puritan Bennett, Pleasanton, CA, USA) to verify the position of the endotracheal tube. Initially, the MUV utilized the Pedi-Cap only when the intubating clinician expressed uncertainty regarding correct tube placement, whereas the LUMC used it routinely.	Following the collaborative video review, MUV providers increased their utilization of the Pedi-Cap to shorten the time to tube position verification and minimize reliance on subsequent auscultation or radiography. As the device was already included in the MUV procedural checklist, this session re-emphasized its clinical utility.
	2	Furthermore, a different brand of face masks had been utilized at the MUV.	After a thorough discussion regarding the pros and cons of the masks employed in Leiden, these models were implemented in Vienna as well.
	3	In addition, disparities in pre-medication regimens for sedation were discussed. The two centers employed different pharmacological agents and reported distinct clinical experiences, prompting a discussion as to why the LUMC opted for propofol while the MUV preferred fentanyl and vecuronium.	No modification was made.
	4	Another procedural variation identified was the method of tube fixation: while the LUMC standardly sutured the tube, the MUV relied solely on adhesive skin strips.	No modification was made.
	5	In both centers, the impact of “human factors” was evident, specifically regarding effective communication and the delivery of correct instructions during video-laryngoscopy-guided intubation.	No modification was made.
	6	Finally, the clinical indicators used to assess adequate sedation differed between the centers: the LUMC evaluated muscle tone in combination with the toe-extension reflex to determine the depth of analgesia, whereas the MUV assessed general infantile muscle tone.	No modification was made.
Session 2: Minimally invasive surfactant therapy/less invasive surfactant administration	7	The primary focus of the multicenter review session on MIST/LISA was the variation in pre-medication regimens utilized prior to surfactant administration. The MUV prioritized non-pharmacological comfort measures, including the administration of sucrose and the utilization of facilitated tucking or nesting. Concurrently, intravenous sedatives—such as ketamine, fentanyl, or midazolam—were administered only on indication. Conversely, the LUMC similarly initiated care with non-pharmacological measures (sucrose and facilitated tucking) but exhibited a lower threshold for escalating to intravenous sedation, standardly utilizing propofol.	Since the multicenter session, the LUMC has placed more emphasis on education and training regarding infant comfort optimization through facilitated tucking during MIST/LISA. This shift was driven by the hypothesis that enhancing non-pharmacological comfort might reduce the subsequent need for pharmacological sedatives. This targeted staff training on comfort measures was subsequently implemented in January 2024.
Session 3: Umbilical venous catheter insertion	8, 9, 12	First, differences in pre-medication were discussed, including differences in prophylactic antibiotics. Second, procedural differences were discussed, including using a purse string suture vs a koperband to prevent bleeding. Finally, antibiotic prophylaxis was routinely administered in Leiden for neonates older than 24 h of life at insertion, whereas no prophylaxis was utilized in Vienna.	No modification was made.
	10	During the UVC insertion session, sterile preparation challenges were discussed. Both centers reported difficulties maintaining umbilical cord sterility. While both institutions utilize a sterilized umbilical cord clamp, clinicians frequently questioned its sterility. The LUMC employed a sterile drape with a central aperture, pulling the cord through with the clamp attached—a technically challenging maneuver. In Vienna, clinicians circumferentially draped multiple smaller sheets around the cord to create an opening, avoiding pulling the clamp through.	Following the session, the LUMC implemented a new cord-cutting technique that eliminated the unsterile clamp from the sterile field. Concurrently, Vienna updated its checklist to include a specific protocol for draping the semi-sterile clamp until cord cleavage.
	11	A procedural variation was also detected in UVC tip localization. The LUMC utilized ultrasonography, whereas the MUV predominantly relied on radiography. Ultrasonography was discussed as a reliable, operator-dependent modality.	Following these discussions, the MUV initiated ultrasound training for junior faculty and implemented a corresponding clinical guideline. Regarding catheterization duration, Vienna enforces a strict seven-day maximum, whereas Leiden evaluates the indication daily; though typically removed by day seven, the LUMC maintains no fixed maximum.
Session 4: Peripherally inserted central catheter (venous lines)	13	During the fourth multicenter session, PICC insertion was discussed, revealing variations in sterile field preparation. The MUV enclosed the neonate’s hands in a sterile bag to prevent contamination from the digits, whereas the LUMC disinfected the hands and placed them directly into the sterile field.	No modification was made.
	14	Additionally, insertion depth was measured differently: either from the insertion site to the target anatomical endpoint (utilized by the MUV) or from the wrist to the axilla (utilized by the LUMC).	No modification was made.
	15	Regarding catheter tip localization, cross-institutional dialogue focused on strategies to introduce ultrasonography as a verification tool and methods to train the clinical team in this emerging technique	Both centers used more ultrasound verification of catheter tip localization after this session.
Session 5: Physiologically based cord clamping	16, 18, 19	In the fifth session, PBCC during stabilization following birth was discussed. Both institutions had recently implemented this technique, revealing variations in the positioning of the Concord Birth Trolley (Concord Neonatal B.V., Leiden, The Netherlands). In the operating room (OR) setting, the MUV placed the trolley platform between the mother’s legs, whereas the LUMC positioned it parallel to the mother. Regarding umbilical cord management, the MUV adhered to a minimum clamping time of two minutes, whereas the LUMC maintained a minimum of three minutes. Both centers reported difficulties visualizing respiratory parameters on the clinical monitor during the procedure. At the MUV, the monitor was positioned behind the proceduralist, whereas the LUMC placed it adjacent to the operator. Strategies were discussed to improve monitor visibility and minimize reliance on verbal team feedback. Finally, the interprofessional discussion highlighted the nurse’s perspective, noting that spatial constraints restricted their mobility, frequently leading to a more passive role and limiting space for essential equipment (e.g., intubation supplies). Consequently, participants emphasized that structured communication and rigorous pre-procedural training are essential to mitigate these spatial limitations.	No modification was made.
	17	Furthermore, the MUV placed the infant in a lateral recumbent position to optimize comfort, whereas the LUMC utilized a supine position. The predominantly utilized delivery modes also differed: the MUV performed PBCC primarily during cesarean sections, whereas the LUMC, whose implementation was ongoing, utilized it mainly during vaginal deliveries.	Based on these review sessions, the LUMC integrated the MUV’s OR setup configuration into its ongoing implementation strategy.

^1^ The number of the identified practice variation corresponds to the numbers used in Figure 2.

## Data Availability

The data presented in this study are available on request from the corresponding author. The data are not publicly available due to privacy and ethical reasons.

## Abbreviations

The following abbreviations are used in this manuscript:

GA	Gestational age
LISA	Less invasive surfactant administration
LUMC	Leiden University Medical Center
MUV	Medical University of Vienna
MIST	Minimally invasive surfactant therapy
NICU	Neonatal Intensive Care Unit
PBCC	Physiologically based cord clamping
PICC	Peripherally inserted venous catheter
RDS	Respiratory distress syndrome
UVC	Umbilical venous catheter
POCUS	Point of care ultrasonography

## Appendix A

**Table A1:** Comparison checklist of the first session: endotracheal intubation.

CHARACTERISTICS	NICU of the MUV	NICU of the LUMC	
GA (birth)	26 + 1	24 + 6	
Birthweight	1160 g	740 g	
Reason for procedure	Pneumothorax	Persistent need for O_2_	
PROCEDURE			Relevance of the identified difference/similarity (Relevant: *) (Not relevant: x)
Catheter size & type	Rüsch, 4 mm	Shiley, 2.5 mm	x
Depth	7 + kg = 10 cm	7 + kg = 8 cm	x
Tube position confirmation	Auscultation, Pedi-Cap if in doubt: chest X-ray	Pedi-Cap, auscultation, chest X-ray	*
Nasogastric tube in situ?	Most of the time	Sometimes; yes in this video	X
Suction of nasopharynx	Mostly, when tube is in situ	Mostly when tube is removed. Air/stomach suction has been performed before procedure	x
Backup respiratory support	Neo-Tee, bag-mask	Neopuff, no bag-mask	x
Mask (type)	Anatomical mask, Vyaire; Size 1	Fisher & Paykel masks; size XS	*
Settings	Neopuff; PIP 20-25/PEEP 5-6	Neopuff; PIP 25 cm H_2_O/PEEP 8 cm H_2_O	x
FiO_2_ value	100%	100%	x
Respirator (type) and settings	Servo-N Conventional ventilation	SLE6000	x
Pre-medication and comfort	Fentanyl Vecuronium	Propofol (atropine, but not used in this video)	*
Assessing comfort	Muscle tone, assessed by physician and nurse subjectively	Stretching of the toes is a marker for grade of analgosedation next to general muscle tone and reactivity of the child.	*
Securing tube	Adhesive skin strips	Suturing of the tube is standard	*
Communication	Sometimes difficult to give correct instructions when using the videolaryngoscope	Sometimes difficult to give correct instructions when using the videolaryngoscope	*

**Table A2:** Comparison checklist of the second session: minimally invasive surfactant therapy/less invasive surfactant administration.

CHARACTERISTICS	NICU of the MUV	NICU of the LUMC	
GA (birth)	30 + 6 wks	29 + 6 wks	
Birthweight	1600 g	1020 g	
Reason for procedure	RDS, FiO_2_ > 35%	RDS, dyspnea, FiO_2_ > 30%	
PROCEDURE			Relevance of the identified difference/similarity (Yes, relevant: *) (No, not relevant: X)
Catheter type & size	Surfcath, Vygon, 6F	Surfcath, Vygon, 6F	x
Nasogastric tube in situ	Most of the time; in this video, yes	Sometimes; in this video, yes	x
Pre-procedure suctioning	Not necessarily; in this video, during laryngoscopy	Air/stomach suction can be performed before procedure	x
Backup respiratory support	Neo-Tee, nCPAP	Neopuff, no bag-mask for CPAP or NIPPV	x
Pre-oxygenation target	>94% on nCPAP	Unknown in this video	x
Primary ventilator model	Acutronic Fabian	SLE6000	x
Equipment	Acutronic Videolaryngoscope	Acutronic Videolaryngoscope	x
Preparation checks	Circulatory stable, IV access secured	No signs of pneumothorax, circulatory stable, IV access secured	x
(pre)medication and sedation	Non-pharmacological analgesia preferred (sucrose, facilitated tucking/nesting) if necessary; Ketamine 0.5 mg/kg, Fentanyl 3–5 µg/kg or Midazolam 0.1 mg/kg	(atropine if necessary) Propofol 1 mg/kg (Sometimes sucrose/facilitated tucking)	*
Positioning of head	Neutral, extra layer	No extra layer under head	x
Procedural specifics	Insert catheter until black mark is not seen anymore Prefer oro-endotracheal insertion Assistant will apply surfactant Suction during application, maintain comfort handling	Bring catheter to desired depth If vocal cords are not in open position, you can pull catheter back and wait Laryngoscope can either be removed (more CPAP pressure)/be left in situ during surfactant administration (assessing surfactant reflux)	x
Heart rate	If bradycardic < 100 bpm, desaturation < 80% intermittent positive pressure ventilation	If bradycardic/desaturation, stop procedure; give CPAP with mouth closed	x
Administration of surfactant	200 mg/kg within 1–3 min in small amounts, administer air afterwards	200 mg/kg within 30 s–3 min in small amounts to avoid reflux	x

**Table A3:** Comparison checklist of the third session: venous umbilical catheter insertion.

CASE	NICU of the MUV	NICU of the LUMC	
GA (birth)	34 + 5	28 + 2	
Birthweight	2500	1300	
Reason for procedure	RDS; 100% FiO_2_, PFC	Access issues, no IV possible	
PROCEDURE			Relevance of the identified difference/similarity (Yes, relevant: *) (No, not relevant: X)
Catheter specifications	Vygon 5 fr, 40 cm, 2 Lumen <1500 g Argyle 2.5–3.5 fr/Vygon 4 fr Term Vygon 5 fr	<3500 g: Vygon 3.5 Fr, 1 lumen >3500 g: 5 fr 4 fr 2 lumen 4.5 fr 3 lumen 8 fr for exchange transfusion	x
Calculated insertion depth	((kg×3) + 9)/2 + 1 = 8.2	((kgx3) + 9)/2 = 6.45	x
(pre)medication & sedation	Non-pharmacological analgesia (sucrose, facilitated tucking/nesting)	>24 h pp, prophylaxis; amoxicillin/clavulanic acid Non-pharmacological analgesia (sucrose, facilitated tucking/nesting)	*
Sterility	Yes	Yes	x
Baby’s position	Neutral	Neutral	x
Medical staff	1–2 experienced neonatal residents, nurse	Experienced pediatric resident, nurse	x
Technique	Disinfection UC/skin/umbilical clamp Sterile draping, flush UVC line Cut umbilical clamp Cut UC and probe the lumen Insert catheter and push to calculated position Fixation by suture	Sterile draping, flush UVC Disinfect umbilical cord and skin with chlorhexidine, clean skin around umbilicus with saline Place a purse string suture around umbilical cord Cut UC at 0.5–1 cm from insertion. Insert catheter and push to calculated position If available, check position by ultrasound Fixation by suture, leukoplast	*
	If a resistance is noticeable/no further push is possible, a second catheter can be pushed forward as well/use pressure on liver to move catheter forward Position/ultrasound guided—if nothing works pull catheter back to peripheral position	If a resistance is noticeable/no further push is possible, a second catheter can be pushed forward next to the first one as well/use pressure on liver to move catheter/ultrasound-guided—if nothing works, pull catheter back to peripheral position	X
Imaging guidance	Always X-ray and, if unclear, ultrasound	If correct position confirmed by ultrasound, no X-ray; recheck position after 1–2 days by ultrasound If position is not confirmed by ultrasound, use X-ray	*
Line duration and maintenance	7 days maximum duration Heparin >36 GA, 1–3 days 150 IE/kg/d after 3 days 300 IE/kg/d 32–36 GA, after 3 days 150 IE/kg/d <32 GA, after 7 days 150 IE/kg/d	The indication for a line is evaluated daily and removed on day 7 This can be deviated from on a case-by-case basis after careful consideration 4 h care in supine for risk of bleeding No maximum duration of stay of catheter Heparin: unnecessary in UVC	*

**Table A4:** Comparison checklist of the fourth session: peripherally inserted central catheter (venous lines).

CASE	NICU of the MUV	NICU of the LUMC	
GA (birth)	29 + 2 GA, 2 days	41 + 5 GA	
Birthweight	1150	3883	
Reason for procedure	Parenteral nutrition	Listeria meningitis, long-term antibiotic administration CVC in arm	
PROCEDURE			Relevance of the identified difference/similarity (Yes, relevant: *) (No, not relevant: X)
Catheter brand & size	Premicath (Vygon, 20 cm, 1 Fr)	Nutriline (Vygon, 30 cm, 2 Fr)	x
Anatomical landmark/target vein	Median cubital vein	Basilic vein	x
Pre-medication & analgesia	Non-pharmacological (sucrose, facilitated tucking, nesting)	Non-pharmacological (sucrose, facilitated tucking, nesting)	x
Sterility & room setup	Strict sterile field; nurse positioned opposite the proceduralist to maintain infant comfort	Strict sterile field; nurse positioned opposite the proceduralist to maintain infant comfort	x
Medical personnel	1–2 clinicians (Neonatologist or Fellow, Nurse)	1–2 clinicians (Neonatologist, Fellow, or Physician Assistant; Nurse)	x
Insertion depth measurement	Site of insertion to the shoulder line	Site of insertion to the shoulder line	x
Specific skin preparation techniques	Hand placed inside a sterile plastic bag and secured with Steri-Strips; entire arm washed and draped	Dual-stage disinfection: non-sterile skin disinfection by the nurse, followed by sterile chlorhexidine prep (allowed to air-dry); sterile draping	*
Vein visualization method	Transillumination utilizing a WeeSight LED device	Transillumination utilizing an Astodia light source	x
Venipuncture & catheter deployment	Apply upper arm tourniquet Puncture target vein and advance catheter Secure catheter using Steri-Strips and Tegaderm transparent dressing	Secure contralateral arm in reactive term infants Apply upper arm tourniquet Introduce introducer needle into the vein Advance PICC using dedicated forceps Withdraw introducer needle Secure with Steri-Strips (optional Grip-Lok and Tegaderm)	*
Position confirmation	Exclusively verified via chest/abdominal X-ray	Primary verification via X-ray or ultrasound; mandated position recheck on Days 1–3 using ultrasound (preferred) or repeat X-ray	*
Continuous heparinization & patency maintenance	Postnatal age & GA-dependent protocol: 50 IU/kg/day adjusted by age milestones: GA > 37 weeks: Initiate on Day 1GA: Initiate Day 3GA <: Initiate Day 7	Weight-dependent continuous infusion concentration: Weight < 1 kg: at Weight > 1 kg: at	x

**Table A5:** Comparison checklist of the fifth session: physiologically based cord clamping at birth.

CASE	NICU of the MUV	NICU of the LUMC	
GA (birth)	29 + 1 GA	31 + 2 GA	
Birthweight	1080	1400	
Reason for Procedure	Cervix insufficiency and preterm labor	Hodgkin lymphoma; from GA 15 start chemotherapy; preterm labor	
PROCEDURE			Relevance of the identified difference/similarity (Yes, relevant: *) (No, not relevant: X)
Brand of Birth Trolley	Concord Birth Trolley	Concord Birth Trolley	x
SYSTEM & ELIGIBILITY			
Bedside Stabilization System	Concord Birth Trolley	Concord Birth Trolley	x
Gestational Age Threshold	<weeks (if no major complications anticipated)	<weeks	x
Absolute Contraindications	TTTS/TAPS, triplets or higher-order multiples	Placenta previa, placenta accreta/percreta, untreated TTTS, monoamniotic/monochorionic twins, triplets or higher-order multiples	x
Cord Clamping Criteria			
Physiological Target Parameters	Spontaneous breathing with target, and HR > 100 bpm	Spontaneous breathing with target, and HR > 100 bpm	x
Clamping Duration (Stable Infant)	Minimum 2 min; maximum 10 min	Minimum 3 min; maximum 10 min	*
Non-Breathing-Infant Strategy	Initiate CPAP (via Perivent; Benveniste if < 28 weeks) and PPV if required. If acute resuscitation or intubation fails on the Concord, transition infant immediately to a standard resuscitation table.	Intubation can be performed directly on the Concord platform (provided parents consent/are comfortable). Once stabilization criteria are achieved, wait 3 min before clamping.	*
Early Clamping Triggers	Maternal: Massive hemorrhage, placental abruption, or obstetrician discretion. Neonatal: Asphyxia/no heart rate requiring immediate advanced resuscitation off the trolley.	Maternal: Massive hemorrhage, placental abruption, or obstetrician discretion. Neonatal: Asphyxia/no heart rate requiring immediate advanced resuscitation off the trolley.	x
Environmental Logistics			
Sterility Protocols	OR: Strict sterility maintained caudal to the umbilical cord; sterile cover applied to Concord platform. DR: Non-sterile procedure.	OR: Neonatologist remains sterile until the pulse oximeter probe is attached; sterile platform cover used. DR: Non-sterile procedure.	x
Spatial Position (OR)	Trolley platform is positioned directly between the mother’s legs. Infant is placed on the platform in a lateral position.	Trolley is positioned parallel to the left side of the operating table. Infant is placed on the platform in a supine position.	*
Spatial Position (DR)	Platform positioned parallel to the mother.	Platform positioned parallel to the mother’s left side near the symphysis pubis; mother’s left leg is lowered to accommodate setup.	*
Medical Personnel	1 Neonatologist, 1 Neonatal Nurse	1 Neonatologist, 1 Neonatal Nurse	x
Procedural workflow			
Standard Steps (OR & DR)	Obstetrician delivers the infant, accounts for umbilical cord length, and transfers the infant to the Concord platform. Avoid touching or manipulating the cord to prevent vasoconstriction/cessation of pulsation. Neonatologist receives the infant, applies a thermal wrap and hat, and attaches a pulse oximeter probe. Once clamping criteria are met, the cord is cut, and the infant is transferred to the NICU.	Obstetrician delivers the infant, accounts for umbilical cord length, and transfers the infant to the Concord platform. Neonatologist receives the infant and applies a thermal wrap and hat, while the nurse attaches the pulse oximeter probe. Maternal oxytocin is administered immediately following cord clamping. Once stabilized, the infant is transferred to the NICU.	*
